# Heat Acclimation Enhances Brain Resilience to Acute Thermal Stress in *Clarias fuscus* by Modulating Cell Adhesion, Anti-Apoptotic Pathways, and Intracellular Degradation Mechanisms

**DOI:** 10.3390/ani15091220

**Published:** 2025-04-25

**Authors:** Yingyi Guan, Cunyu Duan, Xinyu Xie, Zhuoying Luo, Dayan Zhou, Yulei Zhang, Guangli Li, Yu Liao, Changxu Tian

**Affiliations:** 1 Guangdong Research Center on Reproductive Control and Breeding Technology of Indigenous Valuable Fish Species, Guangdong Provincial Engineering Laboratory for Mariculture Organism Breeding, Guangdong Provincial Key Laboratory of Aquatic Animal Disease Control and Healthy Culture, Fisheries College, Guangdong Ocean University, Zhanjiang 524088, China; yingyi_guan@163.com (Y.G.); duancherry77@163.com (C.D.); xie607225@163.com (X.X.); 17325773584@163.com (Z.L.); zhangyl@gdou.edu.cn (Y.Z.); ligl@gdou.edu.cn (G.L.); 2 Guangxi Introduction and Breeding Center of Aquaculture, Nanning 530001, China; magiczdyan@126.com

**Keywords:** catfish, heat acclimation, adaptive regulation, brain transcriptome

## Abstract

Global climate change presents a significant challenge to aquatic ecosystems, with ectothermic fish being particularly sensitive to temperature fluctuations. The brain plays a crucial role in perceiving, regulating, and adapting to thermal changes, and its response to heat stress is crucial for survival. However, the molecular mechanisms underlying heat stress and acclimation in fish brains remain poorly understood. This study aimed to investigate the adaptive mechanisms of Hong Kong catfish (*Clarias fuscus*) brains under heat acclimation and acute heat stress using transcriptome analysis. Fish were divided into two groups: a normal temperature group (NT, 26 °C for 90 days) and a heat-acclimated group (HT, 34 °C for 90 days), followed by acute heat stress (34 °C for 72 h) and recovery (26 °C for 72 h). Heat acclimation improved *C. fuscus* tolerance to acute heat stress, with faster gene responses and stronger neuroprotection. Key pathways enriched included cell adhesion and ECM-receptor interactions during recovery. Apoptosis regulation was balanced, with the HT group upregulating anti-apoptotic genes to mitigate neuronal cell death. Additionally, the lysosome–phagosome pathway was activated during recovery, facilitating the transport of lysosomal enzymes and the clearance of damaged cellular components, aiding neuronal repair. Ribosome biogenesis was suppressed under heat stress to conserve energy, but this suppression was less pronounced in the HT group. In summary, heat acclimation enhances neural protection in *C. fuscus* brains by promoting neuronal repair, suppressing apoptosis, and activating lysosomal pathways, thereby improving tolerance to acute heat stress. These findings offer a molecular basis for breeding heat-tolerant fish species in aquaculture, and deepen our understanding of thermal adaptation in aquatic animals amid global climate change.

## Simple Summary

Climate change has resulted in rising water temperatures, threatening the survival of fish species, such as the Hong Kong catfish. This study aimed to understand how these fish adapt their brains to heat stress. Our findings indicate that fish acclimated to higher temperatures exhibit better brain protection under sudden heat stress. Specifically, their brains rapidly activate genes involved in preventing cell death, repairing tissue, and clearing damaged components. These insights could inform the breeding of more heat-tolerant fish, ensuring sustainable aquaculture practices as global temperatures continue to rise.

## 1. Introduction

With the rapid advancement of global industrialization, climate warming has become an increasingly pronounced trend, resulting in rising atmospheric and water temperatures, as well as an increase in the frequency of extreme climatic events [1]. These environmental changes are having profound effects on aquatic ecosystems, with thermal stress becoming a growing concern in both freshwater and marine environments [2]. As ectothermic organisms, fish are highly sensitive to fluctuations in environmental temperature, with their body temperature directly corresponding to that of the surrounding water [3]. Within an optimal thermal range, fish are capable of maintaining physiological homeostasis through biochemical regulation [4]. However, when water temperatures approach or exceed their thermal tolerance limits, fish experience stress responses that disrupt their physiological balance, which, in extreme cases, can lead to mortality [5]. In aquaculture settings, rising water temperatures have been linked to reduced growth rates, impaired reproductive performance, and increased mortality, all of which negatively impact farming efficiency [6]. Given these challenges, understanding the adaptive strategies of economically important aquaculture species in response to high-temperature conditions is critical for improving their resilience and ensuring sustainable production in the face of increasingly frequent temperature extremes [7].

The fish brain plays a crucial role in perceiving, regulating, and adapting to temperature fluctuations [8]. It maintains physiological homeostasis through multiple mechanisms, including temperature sensing, neuroendocrine regulation, neuroprotection, behavioral modulation via neurotransmitters, and long-term adaptation through gene expression regulation [9]. Temperature fluctuations not only influence the physiological state of neurons but alter neurotransmitter secretion and the activation patterns of brain signaling pathways. For instance, in juvenile common carp (*Cyprinus carpio*), heat stress has been shown to affect neurotransmitter metabolism pathways and modulate the expression of synaptic plasticity-related genes [10]. Similarly, in grass carp (*Ctenopharyngodon idella*), high-temperature stress leads to a significant upregulation of heat shock proteins (HSPs) and calcium homeostasis-related genes in the brain, suggesting that the brain employs enhanced protein homeostasis and neural signaling as adaptive strategies to cope with thermal stress [11]. Moreover, in Senegalese sole (*Solea senegalensis*), the stress-responsive gene *crfbp* was significantly upregulated one week after heat stress, potentially indicating a role in the negative feedback regulation of the hypothalamic–pituitary–interrenal (HPI) axis. However, further studies are needed to clarify its involvement in the long-term adaptation to elevated temperatures [12]. Due to its distinct metabolic demands and highly specialized neuronal populations, the fish brain may adopt unique adaptive strategies under heat stress, different from those observed in other tissues. Despite these observations, the molecular response mechanisms of the fish brain to acute heat stress remain largely unexplored. Furthermore, the ways in which long-term thermal acclimation influences brain heat tolerance are still poorly understood, necessitating further systematic investigation.

In recent years, the advancement of high-throughput sequencing technologies has positioned transcriptomics as a powerful tool for investigating heat stress responses in aquatic organisms [13]. Understanding how fish regulate their physiological responses to acute temperature fluctuations under varying rearing conditions is essential for unraveling their thermal plasticity. Transcriptomic analysis offers a comprehensive approach to exploring gene expression changes in response to environmental stress, allowing for the identification of key signaling pathways involved in stress adaptation. For instance, in turbot (*Scophthalmus maximus*), studies have shown that acute heat stress significantly alters metabolic regulatory networks, particularly affecting glucose and amino acid metabolism in the kidney [14], while lipid metabolism pathways in the liver are also notably modulated [15]. Similarly, in spotted sea bass (*Lateolabrax maculatus*), exposure to acute thermal stress (from 25 °C to 32 °C) results in the upregulation of genes associated with endoplasmic reticulum (ER) protein processing, suggesting the activation of ER stress pathways to maintain protein homeostasis under heat stress. Furthermore, genes associated with the AGE–RAGE signaling pathway were downregulated, potentially reducing oxidative stress and inflammatory responses [16]. In Korean rockfish (*Sebastes schlegelii*), a transcriptomic analysis revealed that acute heat stress induces the activation of the NF-κB pathway through regulation of *ikkalpha* and other related genes, triggering an inflammatory response and upregulating immune-related genes to enhance heat tolerance [17]. These studies highlight that different fish species may adopt distinct molecular strategies to cope with heat stress, reflecting species-specific differences in adaptive response. Overall, transcriptomic analysis provides valuable insights into the molecular mechanisms underlying heat stress responses in fish, offering a genetic foundation for the selective breeding of heat-tolerant strains in aquaculture.

The Hong Kong catfish (*Clarias fuscus*) is a freshwater species widely distributed across Southeast Asia and southern China. It is the only freshwater species within the Clariidae family native to China and has become an economically significant aquaculture species due to its strong environmental adaptability, broad omnivorous diet, and high economic value [18]. However, it remains vulnerable to the impacts of global warming. The optimal temperature range for *C. fuscus* growth is 25–30 °C; however, when water temperature exceeds 35 °C, juvenile mortality rates increase significantly [19]. Previous studies have shown that, under chronic heat stress, *C. fuscus* maintains physiological homeostasis by modulating its immune metabolism [20]. Long-term exposure to heat stress has been found to damage the liver and to alter gene expression patterns in response to acute heat stress [21]. In the gills, heat tolerance is enhanced through structural modifications and increased antioxidant capacity [22]. Furthermore, heat-acclimated *C. fuscus* exhibits improved tolerance to acute heat shock in the head kidney, largely through the regulation of its energy metabolism [23]. Despite these valuable insights, the molecular mechanisms of heat acclimation and acute heat stress responses in the brain—a critical component of the central nervous system (CNS)—remain largely unexplored in *C. fuscus*. Investigating the brain’s response to heat stress will provide important insights into its neural adaptation strategies under extreme temperature fluctuations, helping to better understand the species’ resilience and capacity to thrive under climate change conditions.

This study investigates the adaptive mechanisms of the *C. fuscus* brain under heat acclimation and acute heat stress through transcriptomic analysis. By comparing gene expression changes between control and heat-acclimated groups, we focused on the regulatory patterns of key molecular pathways, including cell adhesion, anti-apoptosis, and intracellular degradation. This study enhances our understanding of the molecular mechanisms underlying neural adaptation to elevated temperatures in fish, and provides theoretical insights for the molecular breeding of heat-tolerant strains and the optimization of aquaculture strategies in the context of climate change.

## 2. Materials and Methods

### 2.1. Ethics Statement

The Animal Research Ethics Committee of Guangdong Ocean University (201903003) approved the experimental protocols for this study [24], in accordance with the Guide for the Care and Use of Laboratory Animals (NIH Pub. No.85-23, revised 1996). This study does not involve any endangered or protected species.

### 2.2. Animals

Juvenile *C. fuscus* used in this experiment were sourced from Guangxi Hongtai Aquaculture Farm, Nanning, China. A total of 2400 juvenile fish were randomly and evenly divided into two groups: one group was kept at 26 ± 2 °C for 90 days (NT group), and the other group was kept at 34 ± 0.5 °C for the same duration (HT group). Each group included three parallel replicates, with 400 fish per replicate. All fish were reared in 700-L plastic circular tanks equipped with continuous aeration using air stones, operating 24 h a day throughout the experiment. Water quality was maintained by replacing one-third of the total water volume daily. Before each water exchange, fresh water was preheated to match the experimental temperature to avoid sudden thermal fluctuations. For the HT group, the initial water temperature was set at 28 °C and gradually increased to 34 °C at a rate of 1 °C per hour using submersible heaters. Once the target temperature was reached, it was maintained at 34 ± 0.5 °C throughout the 90-day rearing period. The water temperature in each tank was monitored twice daily using a digital thermometer to ensure stability. The NT group was maintained at ambient room temperature (26 ± 2 °C). Fish were fed twice daily at 9:00 and 17:00 h. Water quality parameters were monitored every three days throughout the 90-day rearing period to ensure environmental stability. The pH, salinity, and dissolved oxygen (DO) were measured using a ProQuatro multiparameter water quality meter (YSI Inc., Yellow Springs, OH, USA). Ammonia nitrogen (NH₃-N) concentrations were determined using colorimetric assay kits (Fish Doctor^®^, manufactured by Yancheng Bainuo Biotechnology Co., Ltd., Yancheng, China) following the manufacturer’s instructions. Water quality was maintained within optimal ranges for *C. fuscus*, with the following values: pH 6.8–7.5, salinity 0.13 ppt, DO 6.2–7.8 mg/L, and ammonia nitrogen 0.05–0.15 mg/L.

**Figure 1:**
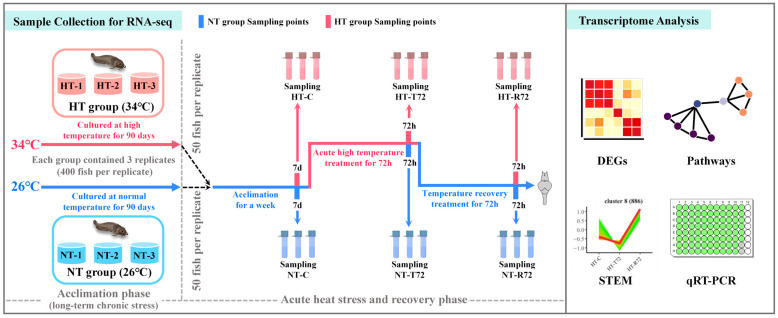
Schematic diagram of temperature treatments at different stages and data analysis workflow for the NT and HT groups.

After 90 days of acclimation, both groups maintained a survival rate of 84%. Subsequently, 50 fish were randomly selected from each replicate (a total of six replicates; body weight: 77.62 ± 6.55 g; body length: 19.01 ± 0.50 cm) to undergo acute high-temperature stress at 34 °C for 72 h, followed by a recovery phase at 26 °C for 72 h, as shown in Figure 1. Brain tissues were collected from six randomly selected fish in both the NT and HT groups at three time points: immediately before acute heat exposure (C), after 72 h of heat stress (T72), and after 72 h of recovery at 26 °C (R72). The collected tissues were immediately preserved in liquid nitrogen at −80 °C for further analysis. Detailed procedures were described in a previous experiment [21].

### 2.3. Brain Transcriptome Analysis

For each group and time point, brain tissues were collected from six fish. Every two samples were pooled to obtain one biological replicate, resulting in three pooled samples per group per time point. These pooled samples were used for RNA extraction and transcriptome sequencing. Total RNA was extracted from brain tissues using TRIzol^®^ Reagent (Invitrogen, Carlsbad, CA, USA), following the manufacturer’s protocol. Genomic DNA was removed using RNase-Free DNase I (Thermo Fisher Scientific, Waltham, MA, USA). RNA concentration and purity were assessed using a NanoDrop™ 2000 spectrophotometer (Thermo Fisher Scientific, Waltham, MA, USA), and RNA integrity was confirmed using an Agilent 2100 Bioanalyzer (Agilent Technologies, Santa Clara, CA, USA). Only RNA samples with RIN > 7.0 were used for library construction. RNA libraries were constructed using the NEBNext^®^ Ultra™ RNA Library Prep Kit (NEB, Ipswich, MA, USA), according to the manufacturer’s protocol. Library quantification was performed using a Qubit 2.0 Fluorometer (Thermo Fisher Scientific, Waltham, MA, USA), and the concentration was adjusted to 1.5 ng/μL. The insert size of the library was evaluated using the Agilent Bioanalyzer 2100. Subsequently, qRT-PCR was conducted to determine the effective concentration of the libraries, ensuring that they met the quality threshold (≥2 nM). Sequencing was conducted on an Illumina NovaSeq 6000 platform (Illumina, San Diego, CA, USA), generating 150 bp paired-end reads. Raw sequencing reads were quality-filtered to remove low-quality bases and adapter sequences. Clean reads were assembled using the Trinity software package (v2.14.0) [25] with default parameters. Paired-end clean reads were then aligned to the reference genome [18] using HISAT2 v2.0.5, and gene annotation was carried out using the gene set from the same published genome [18]. Gene expression quantification was performed using the Subread package 2.1.1, followed by differential expression analysis with DESeq2 1.48.0, applying a significance threshold of padj < 0.05 and |log_2_FoldChange| ≥ 1.0. Gene Ontology (GO) annotation and Kyoto Encyclopedia of Genes and Genomes (KEGG) pathway enrichment analyses were conducted using the clusterProfiler R package (v3.8.1) to identify key biological processes associated with differentially expressed genes (DEGs). RNA-Seq data have been deposited in the NCBI database under the project accession number PRJNA1224955.

### 2.4. Trend Clustering

The temporal dynamics of DEGs were analyzed by assessing their expression patterns over time. Firstly, DEGs from both the NT and HT groups were normalized, and the average expression level of each gene at various time points was calculated. Next, the Elbow Method was applied to determine the optimal number of clusters, ensuring maximal inter-cluster variability while minimizing intra-cluster differences. A Total Within Sum of Squares (WSS) curve was plotted against various cluster numbers (k) for both the NT and HT groups (Figure S1). Based on the curve trend, WSS stabilized when the cluster number exceeded 5 in the NT group, whereas the HT group showed the last significant drop in WSS at k = 8, after which it remained stable. Therefore, k = 8 was chosen as the optimal number of clusters for subsequent trend analysis. Fuzzy c-means clustering analysis was conducted using the Mfuzz R package (v3.8.1) to classify DEGs into distinct temporal expression patterns.

### 2.5. qRT-PCR Validation

To verify the reliability of the RNA-Seq results, qRT-PCR was performed to validate the expression patterns of nine DEGs, which were randomly selected from different functional pathways to avoid potential bias caused by tightly related upstream or downstream regulatory genes. This analysis allows us to confirm the accuracy of the transcriptomic data by assessing the consistency of gene expression trends. The nine genes are listed in Table S1. Total RNA extraction and reverse transcription were performed according to an established protocol [21]. The qRT-PCR analysis was performed using a Roche LightCycler^®^ 480 II Real-Time PCR System (Roche, Basel, Switzerland). The housekeeping gene *actb2* was used as the internal reference, and its primer sequences are listed in Table S1. The expression stability of *actb2* has been previously validated in *C. fuscus*, and it showed the highest stability among several candidate reference genes in our previous publication [26]. The qRT-PCR cycling parameters were initial denaturation at 95 °C for 5 min, 40 cycles of denaturation at 95 °C for 30 s, and annealing/extension at 53 °C for 30 s. The qRT-PCR cycle was performed with a 2^−ΔΔCt^ method. Finally, the relative expression was calculated using the 2^−ΔΔCt^ method to determine the expression changes of the selected genes during the temperature treatment.

## 3. Results

### 3.1. Quality of Transcriptome Sequencing

Each experimental group and time point included three biological replicates (n = 3), resulting in a total of 18 transcriptome samples. In the NT group, the number of clean reads ranged from 39,096,830 to 54,391,062, while in the HT group, the number ranged from 38,316,990 to 53,383,510. The Q20 values for all samples exceeded 96.39%, and the Q30 values ranged from 91.43% to 93.52%, indicating high sequencing quality with low error rates. The GC content analysis revealed that the GC content in the NT group ranged from 41.31% to 46.39%, whereas, in the HT group, it ranged from 42.29% to 47.48%. The unique mapping rate varied from 63.21% to 97.12% in the NT group, and between 76.23% and 88.14% in the HT group (Tables S2 and S3). These results confirm that the sequencing data generated in this study exhibit high quality, and meet the criteria for subsequent analyses.

### 3.2. Analysis of DEGs

**Figure 2:**
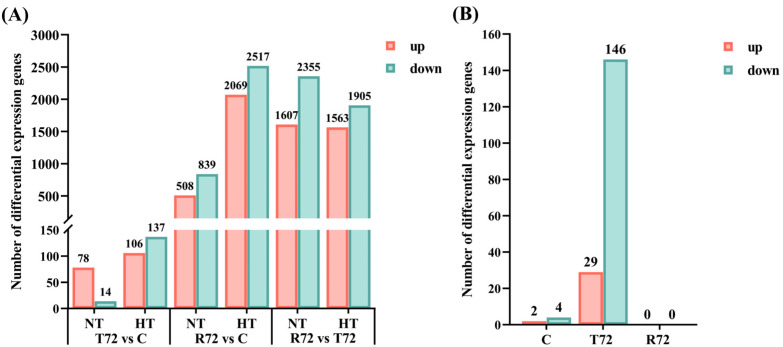
(**A**) The number of DEGs produced by the comparison of the two groups at different treatment periods. (**B**) The number of DEGs generated between the two groups at the same treatment period.

Differential expression analysis using DESeq2 identified a total of 10,386 DEGs, including 4443 in the NT group and 5943 in the HT group. In the NT group, comparisons between T72 vs. C, R72 vs. C, and R72 vs. T72 revealed 78, 508, and 1067 upregulated genes, and 14, 839, and 2355 downregulated genes, respectively. In the HT group, the corresponding comparisons identified 106, 2069, and 1563 upregulated genes, and 137, 2517, and 1905 downregulated genes (Figure 2A). When comparing the HT and NT groups, at the C and T72 stages, 2 and 29 genes were upregulated, and 4 and 146 genes were downregulated, respectively. No DEGs were detected at the R72 stage (Figure 2B).

**Figure 3:**
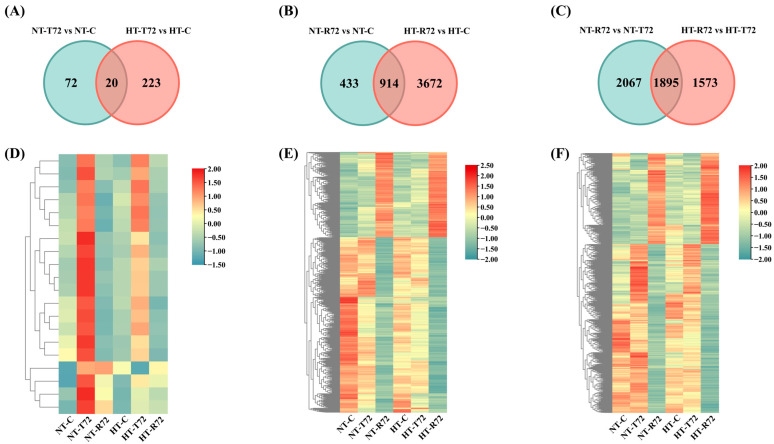
(**A**) Overlap of DEGs between NT-T72 vs. NT-C and HT-T72 vs. HT-C. (**B**) Overlap of DEGs between NT-R72 vs. NT-C and HT-R72 vs. HT-C. (**C**) Overlap of DEGs between NT-R72 vs. NT-T72 and HT-R72 vs. HT-T72. (**D**) Hierarchical clustering analysis of shared DEGs in NT-T72 vs. NT-C and HT-T72 vs. HT-C. (**E**) Hierarchical clustering analysis of shared DEGs in NT-R72 vs. NT-C and HT-R72 vs. HT-C. (**F**) Hierarchical clustering analysis of shared DEGs in NT-R72 vs. NT-T72 and HT-R72 vs. HT-T72.

To further investigate DEGs across different treatment stages and between groups, a Venn diagram analysis was conducted (Figure 3A–C). During the acute heat stress phase (T72 vs. C), the number of shared DEGs between the NT and HT groups was relatively low (20 genes), with the NT group showing 72 unique DEGs and the HT group exhibiting 223 unique DEGs. This suggests that heat acclimation has a distinct regulatory effect on gene expression in *C. fuscus*. During the temperature recovery phase (R72 vs. C), the number of shared DEGs between the two groups increased to 914 genes. However, the HT group still exhibited a strong specific regulatory effect, as evidenced by the 433 unique DEGs in the NT group and the 3672 unique DEGs in the HT group. A heatmap analysis (Figure 3D–F) further revealed the expression patterns of the shared DEGs. The response patterns of these genes to acute heat stress were similar in both groups, but the magnitude of gene expression changes was greater in the NT group. To provide detailed information, the complete list of DEGs for each comparison group is included in Supplementary Tables S4–S9, and an extended heatmap displaying all DEGs is presented in Supplementary Figure S2.

### 3.3. Functional and Pathway Enrichment Analysis of DEGs

**Figure 4:**
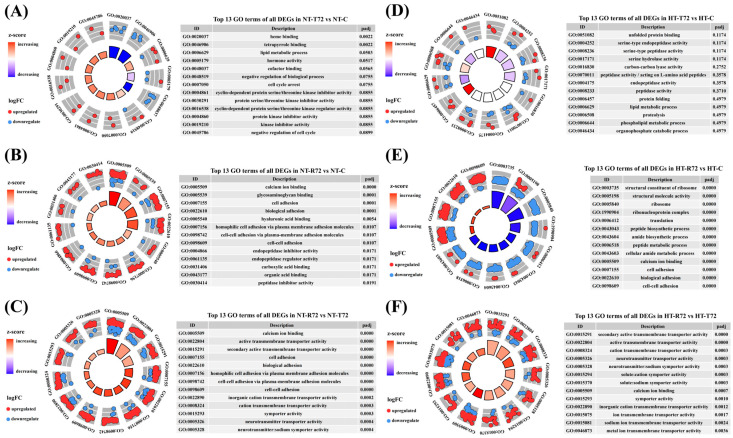
GO Enrichment Analysis of DEGs in Each Comparison Group. The inner ring represents the enrichment significance of each GO term, with bar height decreasing from inside to outside, indicating decreasing enrichment significance. The bars are color-coded based on the Z-score, where red represents upregulated enrichment, blue represents downregulated enrichment, and the color gradient from red to blue reflects a decreasing Z-score. The outer ring displays DEGs distributed according to their log₂FC values, where red dots represent upregulated genes and blue dots represent downregulated genes. (**A**–**C**) represent NT-T72 vs. NT-C (**A**), NT-R72 vs. NT-C (**B**), and NT-R72 vs. NT-T72 (**C**) comparisons, while (**D**–**F**) correspond to HT-T72 vs. HT-C (**D**), HT-R72 vs. HT-C (**E**), and HT-R72 vs. HT-T72 (**F**) comparisons.

To investigate the impact of long-term heat acclimation on the brain’s response to temperature fluctuations in *C. fuscus*, GO and KEGG enrichment analyses were conducted for DEGs across different treatment groups. In the NT group (Figure 4A–C), a GO enrichment analysis revealed distinct patterns at different treatment stages. After acute heat stress (NT-T72 vs. NT-C), DEGs were significantly enriched in heme binding and tetrapyrrole binding, both of which were predominantly downregulated. Following temperature recovery (NT-R72 vs. NT-C), DEGs were significantly enriched in calcium ion binding, glycosaminoglycan binding, cell adhesion, and biological adhesion, showing overall upregulation. In the NT-R72 vs. NT-T72 comparison, enriched GO terms were mainly associated with calcium ion binding, active transmembrane transporter activity, secondary active transmembrane transporter activity, and cell adhesion. By contrast, the HT group (Figure 4D–F) displayed distinct enrichment patterns. After acute heat stress (HT-T72 vs. HT-C), no significantly enriched GO terms were observed. Following temperature recovery (HT-R72 vs. HT-C), DEGs were enriched in ribosomal structural components and translation-related processes, with a trend of downregulation. In the HT-R72 vs. HT-T72 comparison, DEGs were significantly enriched in transmembrane transporter activity terms related to neural activity, with overall upregulation.

**Figure 5:**
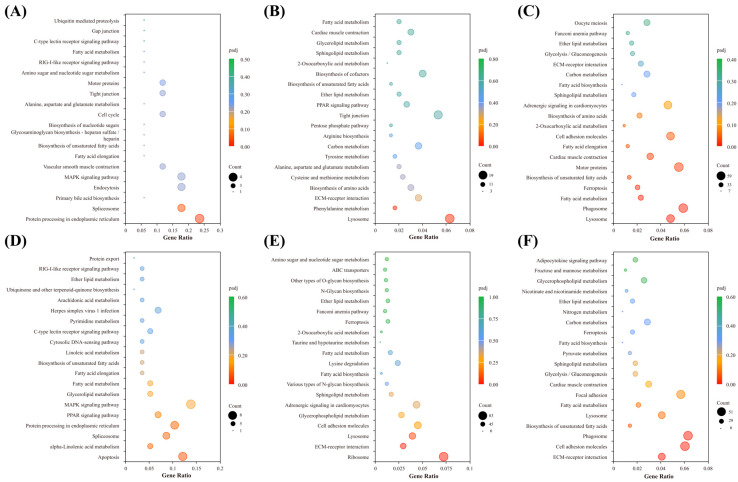
KEGG Pathway Enrichment Analysis of DEGs in Each Comparison Group. (**A**–**C**) represent NT-T72 vs. NT-C (**A**), NT-R72 vs. NT-C (**B**), and NT-R72 vs. NT-T72 (**C**) comparisons, while (**D**–**F**) correspond to HT-T72 vs. HT-C (**D**), HT-R72 vs. HT-C (**E**), and HT-R72 vs. HT-T72 (**F**) comparisons.

A KEGG pathway enrichment analysis identified metabolic and signaling pathways associated with DEGs under different temperature treatments (Figure 5). In the NT group (Figure 5A–C), after acute heat stress, DEGs were significantly enriched in the protein processing in the endoplasmic reticulum and the spliceosome pathway. Following temperature recovery, DEGs were significantly enriched in the lysosome and phenylalanine metabolism pathways. In the NT-R72 vs. NT-T72 comparison, DEGs were predominantly enriched in pathways related to lysosome, phagosome, fatty acid metabolism, and ferroptosis. In the HT group (Figure 5D–F), DEGs during the acute heat stress phase were primarily enriched in the apoptosis pathway. During temperature recovery, DEGs were primarily enriched in the ribosome, lysosome, and extracellular matrix (ECM)-receptor interaction pathways. In the HT-R72 vs. HT-T72 comparison, DEGs remained enriched in the ECM-receptor interaction pathway, along with the phagosome and cell adhesion molecule pathways.

### 3.4. Trend Clustering Analysis

**Figure 6:**
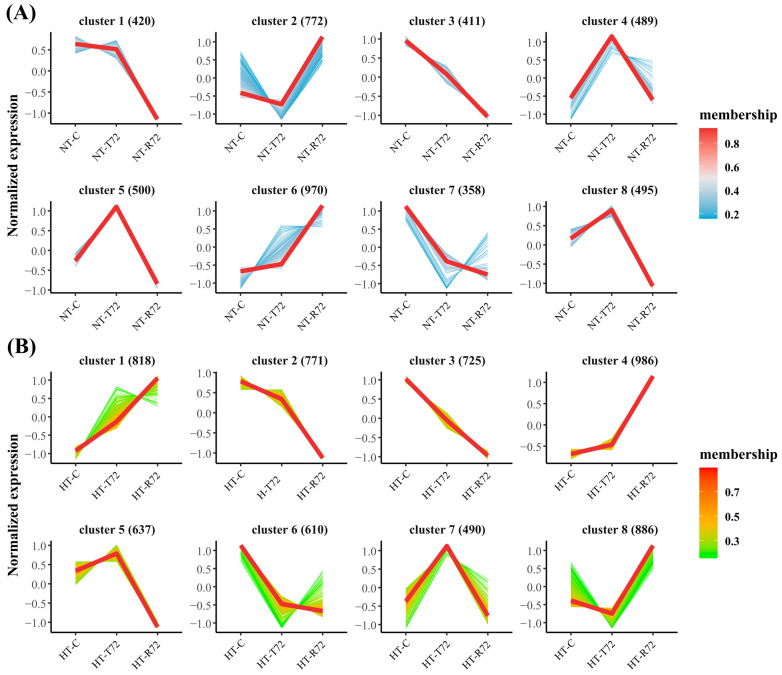
Trend clustering analysis of DEGs in the NT group (**A**) and HT group (**B**). DEGs in both groups were classified into eight distinct expression patterns (clusters 1–8). The y-axis represents normalized gene expression levels, while the x-axis indicates different treatment stages. The color of the curves represents gene membership, with red indicating higher membership values and blue (**A**) or green (**B**) indicating lower membership values.

**Figure 7:**
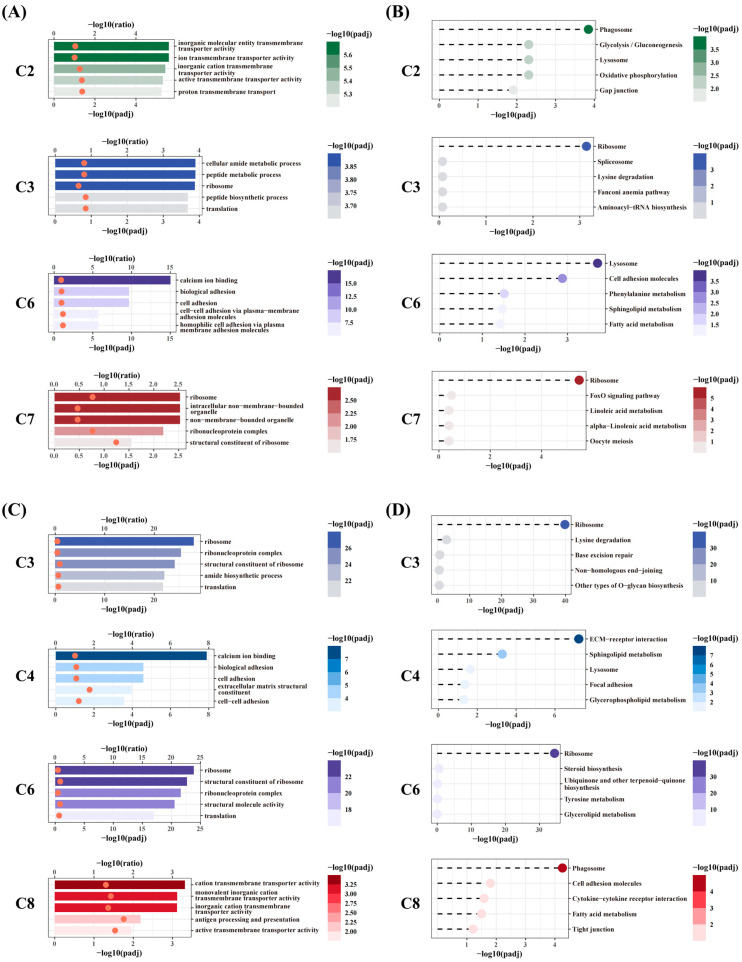
GO and KEGG enrichment analysis of selected gene clusters after trend clustering in the NT group (**A**,**B**) and HT group (**C**,**D**). (**A**,**C**) GO functional enrichment analysis. The y-axis represents GO terms. The upper x-axis indicates −log10(ratio), with its value represented by the position of the orange dots. The lower x-axis indicates −log10(padj), with bar color intensity reflecting enrichment significance, where darker colors denote higher significance. (**B**,**D**) KEGG pathway enrichment analysis. The color intensity of the dots represents −log10(padj), with darker colors indicating higher enrichment significance.

A trend clustering analysis revealed distinct gene expression patterns among the DEGs in the NT and HT groups, resulting in eight clusters (C1–C8). In the NT group (Figure 6A), the gene counts for clusters C1–C8 were 420, 772, 411, 489, 500, 970, 358, and 495, respectively. In the HT group (Figure 6B), the corresponding counts were 818, 771, 725, 986, 637, 610, 490, and 886. GO and KEGG enrichment analyses were performed for each cluster in both groups to uncover the functional significance of genes exhibiting specific expression trends (Figure 7 and Figure S3).

In the NT group, genes in C3 and C7 exhibited sustained downregulation following acute heat stress, failing to return to normal levels even after rewarming. A GO enrichment analysis revealed that C3 genes were primarily associated with ribosomal function and protein metabolism, while C7 genes were enriched in cellular organelles and structural components (Figure 7A). A KEGG pathway analysis further confirmed that both clusters were significantly enriched in the ribosome pathway, highlighting a heat-induced inhibition of ribosome biogenesis (Figure 7B). Conversely, genes in C2 and C6 peaked in expression during the rewarming phase, suggesting their potential involvement in neural repair mechanisms. A GO analysis indicated that C2 was enriched in transmembrane transporter activity, while C6 was significantly associated with calcium ion binding and cell adhesion. A KEGG enrichment analysis further showed that C2 was significantly enriched in the phagosome pathway, whereas C6 was highly associated with the lysosome pathway, suggesting their roles in cellular degradation and repair processes.

In the HT group, the expression patterns of C3 and C6 closely resembled those of C3 and C7 in the NT group, but with a larger number of genes involved. A GO analysis confirmed that genes in C3 and C6 (HT group) were all enriched in ribosomal structural components and translation-related processes (Figure 7C), and a KEGG pathway analysis consistently placed them in the ribosome pathway (Figure 7D). Genes in C4 and C8 exhibited a sharp increase during the rewarming phase. A GO analysis showed that C4 genes were significantly enriched in calcium ion binding and extracellular matrix (ECM) structure, while C8 genes were predominantly associated with transmembrane transporter activity and antigen processing and presentation. A KEGG enrichment analysis revealed that C4 was significantly enriched in the ECM-receptor interaction pathway, whereas C8 was primarily associated with the phagosome pathway.

### 3.5. Molecular Pathway Alterations in the Brain Under Heat Stress

**Figure 8:**
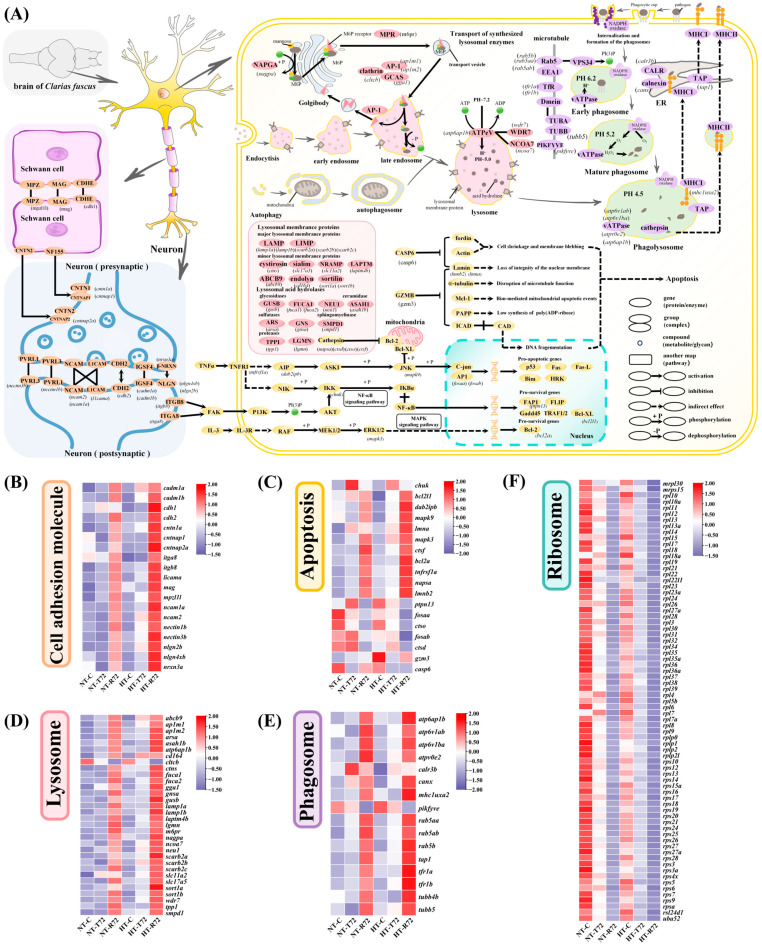
(**A**) Key pathways with significant changes under the experimental conditions (partial list). Orange represents the cell adhesion molecule (CAM) pathway. Yellow represents the apoptosis-related pathways. Pink represents the lysosomal function pathway. Purple represents the phagosome function pathway. (**B**) Heatmap of DEGs in the cell adhesion molecule pathway. (**C**) Heatmap of DEGs in the apoptosis pathway. (**D**) Heatmap of DEGs in the lysosome pathway. (**E**) Heatmap of DEGs in the phagosome pathway. (**F**) Heatmap of DEGs encoding ribosomal large and small subunit components.

Under different temperature treatments, several key pathways in the brain of *C. fuscus* exhibited significant changes in gene expression, particularly those associated with apoptosis, lysosome, phagosome, ribosome, and cell adhesion molecules (Figure 8). We further summarized the detailed information on these differentially expressed genes, including gene IDs, chromosomal locations, functional annotations, log₂ fold changes, and adjusted *p*-values, to facilitate interpretation (Table S10).

In the apoptosis pathway, several genes involved in cellular stress responses and apoptosis inhibition, such as *ptpn13*, *fosaa*, *ctso*, *fosab*, and *ctsd*, showed elevated expression during heat stress, but were downregulated after rewarming, with overall lower expression in the HT group compared to the NT group (Figure 8C). During rewarming, genes such as *bcl2l1*, *bcl2a*, *dab2ip*, *tnfrsf1a*, *mapk3*, *mapk9*, *napsa*, *lmna*, and *lmnb2*, which are linked to apoptosis inhibition and nuclear integrity, were overexpressed, with significantly higher levels in the HT group than in the NT group.

In the lysosome pathway, genes associated with lysosomal structure and function were significantly upregulated during rewarming, with higher expression levels in the HT group (Figure 8D). These included lysosomal membrane proteins (*lamp1a*, *lamp1b*, *scarb2a*, *scarb2b*, *scarb2c*, *slc17a5*) and lysosomal acid hydrolases (*gusb*, *fuca1*, *neu1*, *arsa*, *tpp1*, *lgmn*). Autophagy-related genes, such as *lapmt4b* and *abcb9*, were also highly expressed in the HT group. Genes involved in lysosomal enzyme transport, acidification, and regulation were upregulated during rewarming, except for *cltcb*, which was downregulated after acute heat stress, and remained low. In the phagosome pathway, most genes were significantly upregulated during rewarming, with higher overall expression in the HT group (Figure 8E). For example, genes involved in early endosome formation and phagosome maturation, like *rab5b* and *rab5a*, were upregulated. Tubulin genes (*tubb4b*, *tubb5*), which facilitate phagosome movement and fusion with lysosomes, also showed increased expression. Additionally, genes associated with antigen presentation after phagosome degradation, including *tap1* and *mhc1uxa2*, were upregulated during the rewarming phase.

In the cell adhesion molecule (CAM) pathway, multiple genes were significantly upregulated during the rewarming phase, with higher expression levels observed in the HT group (Figure 8B). These genes include those involved in axon myelination, such as *mpz11l*, *mag*, and *cdh1*; genes that mediate adhesion between Schwann cells and neuronal axons, including *cntn1a*, *cntnap1*, and *cntnap2a*; and genes that facilitate direct adhesion between neurons, such as *ncam1a*, *ncam2*, *l1cama*, *nectin1b*, *nectin3b*, *cdh2*, and *cadm1a*. In the ribosome pathway, ribosomal component genes were highly expressed under normal temperatures, exhibited a uniform decline during acute heat stress, and remained significantly downregulated in the rewarming phase, with overall lower expression levels in the HT group compared to the NT group (Figure 8F). These genes primarily belong to the rpl and rps gene families, encompassing the major structural components of both the large and small ribosomal subunits.

### 3.6. qRT-PCR Validation

**Figure 9:**
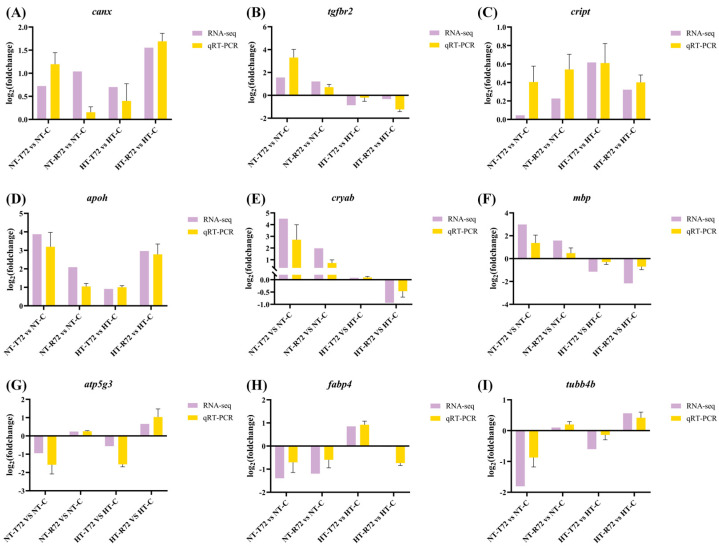
Comparative expression analysis based on RNA-seq and qRT-PCR results. Each bar represents the mean ± SD of nine values obtained from three biological replicates, with each replicate measured in triplicate. (**A**) Expression of *canx*; (**B**) Expression of *tgfbr2*; (**C**) Expression of *cript*; (**D**) Expression of *apoh*; (**E**) Expression of *cryab*; (**F**) Expression of *mbp*; (**G**) Expression of *atp5g3*; (**H**) Expression of *fabp4*; (**I**) Expression of *tubb4b*.

Nine genes (*canx*, *tgfbr2*, *cript*, *apoh*, *cryab*, *mbp*, *atp5g3*, *fabp4*, and *tubb4b*) were selected for qRT-PCR analysis. The qRT-PCR results confirmed that the expression patterns of these genes were consistent with the RNA-seq data across different treatments (Figure 9). This validation indicates that the gene expression trends observed in the RNA-seq data are accurate and reliable.

## 4. Discussion

This study investigated the adaptive mechanisms of the *C. fuscus* brain under heat acclimation and acute heat stress using transcriptomic analysis. The results indicate that heat-acclimated fish exhibit a more rapid gene response, enhanced anti-apoptotic capacity, and more active cellular repair mechanisms, suggesting that long-term heat adaptation confers increased thermal tolerance. A particularly noteworthy finding was that the brain of *C. fuscus* adopted an anti-apoptotic strategy in response to acute heat stress, a response that contrasts with those observed in other tissues. Pro-apoptotic genes were downregulated, while anti-apoptotic genes were upregulated, potentially contributing to neuronal survival and the stabilization of the neural system. This pattern diverges from the typical apoptotic responses seen in other tissues under heat stress, suggesting that the brain utilizes a more conserved neuroprotective strategy to withstand acute thermal challenges. The following discussion will focus on the neuroprotective mechanisms of *C. fuscus* under heat stress, the role of heat acclimation in enhancing thermal tolerance, and the regulation of ribosome biogenesis and protein synthesis in maintaining cellular function under thermal stress.

### 4.1. Enhanced Neural Resilience Through Accelerated Activation of Cell Adhesion and ECM Pathways

Cell adhesion molecules (CAMs) play a crucial role in maintaining connections between Schwann cells, neurons, and the myelin sheath, regulating synaptic connectivity, myelination, and neural repair [27]. In this study, a KEGG enrichment analysis revealed significant enrichment of CAMs in the HT group during both the high-temperature phase and the rewarming phase. Similar findings have been reported in other species under heat stress, such as turbot (*Scophthalmus maximus*) and yamame (*Oncorhynchus masou*) [28,29], suggesting that the regulation of CAMs may serve as a strategy for fish to maintain tissue structural stability under thermal stress. In the brain, CAMs not only contribute to structural integrity but play critical roles in neural functions. For instance, *mpzl1l* maintains myelin sheath stability and enhances neural impulse conduction [30], while *cdh1*, which encodes classical E-cadherin, Figure 7 forms tight junctions between Schwann cells, preserving myelin integrity and neural functions [31]. Additionally, *mag* contributes to axon adhesion and recognition, promoting neuronal survival and regeneration [32]. In this study, both *mpzl1l* and *mag* were significantly upregulated during the recovery phase following acute heat stress, with expression levels notably higher in the HT group compared to the NT group. These findings suggest that *C. fuscus* exhibits transcriptional upregulation of cell adhesion-related genes following heat stress, contributing to neural structural stability. Notably, while *cdh1* showed no significant changes in the NT group, it was significantly upregulated in the HT group during recovery, indicating that heat acclimation may reinforce neuroprotection, enabling a faster and more stable response to thermal stress.

In neuron–Schwann cell interactions, *cntn1a* is essential for axon myelination, promoting axon stability and myelin formation [33]. *Cntnap1*, encoding contactin-associated protein, cooperates with *cntn1a* to stabilize myelin segment organization [34]. Meanwhile, *cntnap2a* facilitates axon–Schwann cell interactions, ensuring myelin–node connectivity and efficient nerve impulse transmission [35]. In this study, *cntn1a*, *cntnap1*, and *cntnap2a* were significantly upregulated during the recovery phase following acute heat stress, with higher expression levels observed in the HT group compared to the NT group. These results suggest that heat-acclimated *C. fuscus* may enhance neuron–Schwann cell adhesion, mitigating heat-induced damage to myelin and neural conduction. In neurons, *nectin3b* and *nectin1b* encode Nectin-class adhesion molecules essential for synapse formation and maintenance, particularly at pre- and postsynaptic sites [36]. Similarly, *nrxn3a*, *nlgn2b*, and *nlgn4xb* encode synaptic adhesion proteins that form trans-synaptic bridges, stabilizing synaptic connections [37]. Other adhesion molecules, including *cadm1a*, *cadm1b*, *cdh2*, *ncam1a*, *ncam2*, and *l1cama*, contribute to neural circuit stability and synaptic plasticity. Notably, *l1cama* plays a key role in axon guidance, synapse formation, and neuronal migration, and its upregulation during recovery suggests a role in neuron repair after heat stress [38]. In this study, these genes were highly expressed during the recovery phase, with the HT group showing upregulation as early as the acute heat stress phase. This suggests that heat acclimation accelerates the transcriptional response, facilitating thermal adaptation.

In addition to cell adhesion molecules, we observed that integrin family genes, *itga8* and *itgb8*, were upregulated in neuronal cell membranes under heat stress. Integrins interact with the extracellular matrix (ECM) to regulate cytoskeletal dynamics and activate PI3K/AKT and MAPK/ERK signaling pathways, enhancing anti-apoptotic responses and promoting cellular repair [39]. A KEGG enrichment analysis revealed significant upregulation of the ECM-receptor interaction pathway in the HT group, with integrin-related genes and ECM components, such as laminins and collagens, showing marked upregulation. This suggests that heat acclimation may facilitate ECM-mediated neuroprotection, improving the brain’s resilience to thermal stress. A similar adaptive strategy has been reported in grass carp (*Ctenopharyngodon idella*), where a transcriptome analysis at 34 °C revealed significant upregulation of integrins, suggesting their involvement in regulating cell survival and stress responses through ECM-receptor signaling pathways [40]. Similarly, in yamame, the expression levels of ECM-related genes, including collagens, integrins, and laminins, were upregulated under high-temperature conditions [29]. Notably, our trend analysis showed that cluster 4 in the HT group exhibited a pattern consistent with temperature changes, and a KEGG enrichment analysis of this cluster revealed significant enrichment in the ECM-receptor interaction pathway. This suggests a strong association between this pathway and heat stress responses. By contrast, such a pattern was not observed in the NT group, further indicating that heat acclimation may accelerate the transcriptional response of ECM-related genes to cope with acute heat stress.

Overall, cell adhesion molecules and ECM-receptor interactions play a critical role in neural repair and structural reconstruction, promoting cell–cell adhesion and interaction to restore brain tissue functionality. The enhanced expression of these genes in the HT group suggests a transcriptional response potentially associated with enhanced neural recovery following heat stress.

### 4.2. Dual Regulation of Cell Death and Degradation Pathways Supports Neuroprotection Under Heat Stress

Apoptosis is a genetically regulated process that eliminates damaged cells, preventing further harm to surrounding tissues under environmental stress [41,42]. However, neurons, as highly differentiated cells, have limited regenerative capacity. Compared to other tissues, neuronal apoptosis more readily reaches a threshold where tissue function is compromised, making neurons more vulnerable to excessive cell death [43]. Thus, suppressing unnecessary apoptosis is essential for maintaining neural functions during acute heat stress. Our study revealed that heat acclimation modulates both the JNK/AP-1 pro-apoptotic pathway and the PI3K/AKT anti-apoptotic pathway, which may help mitigate heat-induced neuronal damage and contribute to neuroprotection.

Under heat stress, the JNK signaling pathway plays a central role in neuronal apoptosis in fish [44]. In this study, we observed differential expression of key genes in this pathway. *Tnfrsf1a* mediates TNF-α/TNFR1 signaling, leading to the activation of JNK (*mapk9*), which subsequently phosphorylates c-Jun and induces the AP-1 transcription factor complex (*fosaa*, *fosab*). This complex, in turn, induces the expression of downstream pro-apoptotic genes, including *p53*, *Fas*, *Fas-L*, *Bim*, and *HRK* [45,46]. Additionally, cathepsins, a group of lysosomal proteases, may contribute to apoptosis through lysosomal leakage [47]. However, in the *C. fuscus* brain, key genes involved in JNK/AP-1-mediated apoptosis exhibited relatively low expression, and were further downregulated in the HT group. This suggests that heat acclimation may enhance anti-apoptotic capacity as an adaptive response to high temperatures. Previous studies on Chinese tongue sole (*Cynoglossus semilaevis*) have found that the JNK signaling pathway is rapidly activated in response to temperature changes, regulating neuronal apoptosis [48]. Our results demonstrated that focal adhesion kinase (FAK) activates PI3K through integrin signaling, which subsequently activates AKT, enhancing NF-κB signaling and promoting the expression of the Bcl-2 family anti-apoptotic factors. Notably, *chuk*, *bcl2a*, and *bcl2l1* were significantly upregulated in the acute heat stress phase of the HT group, suggesting that NF-κB activation occurs early during heat acclimation, contributing to enhanced neuronal survival [49]. A similar upregulation of the PI3K/AKT pathway was observed in Siberian sturgeon (*Acipenser baerii*) under chronic heat stress, where it helped reduce apoptosis [50]. These results indicate that heat-acclimated *C. fuscus* may employ a dual regulatory strategy—suppressing JNK/AP-1-mediated apoptosis while activating the PI3K/AKT survival pathway—to strengthen neuroprotection under thermal stress.

Despite the suppression of apoptosis reducing neuronal damage, heat stress-induced metabolic waste and damaged proteins must be promptly cleared to maintain neural homeostasis [51]. In this study, we observed the differential expression of genes associated with lysosome and phagosome pathways in the *C. fuscus* brain following heat stress. This may represent a compensatory clearance mechanism for apoptosis, potentially contributing to neuronal repair [52]. As the intracellular degradation center, the lysosome facilitates the removal of damaged proteins and organelles, preventing their accumulation and subsequent neurotoxicity [53]. Notably, *m6pr*, *ap1m1*, *ap1m2*, and *gga1* were significantly upregulated during the rewarming phase in the HT group, indicating enhanced lysosomal enzyme transport, which may contribute to an increased lysosomal degradation capacity. A similar mechanism has been reported in pikeperch (*Sander lucioperca*) and Atlantic salmon (*Salmo salar*), where heat stress upregulated lysosomal acid hydrolases, improving autophagic efficiency and supporting cellular homeostasis [54,55]. Furthermore, upregulation of the cytoskeleton-related genes *tubb4b* and *tubb5* following heat stress may facilitate phagosome transport and lysosome fusion in *C. fuscus*, potentially contributing to the clearance of damaged components [56]. Interestingly, our results indicate that heat stress may enhance antigen presentation in the *C. fuscus* brain. The degradation products from lysosomes and phagolysosomes can be processed via MHCI and MHCII molecules and presented through TAP, thereby triggering a specific immune response [57,58]. We found that *mhc1uxa2*, *canx*, and *tap1* were significantly upregulated immediately after acute heat stress in the HT group, reaching peak expression during the rewarming phase. This suggests that heat acclimation may influence immune-related gene expression in the *C. fuscus* brain, consistent with the findings for grass carp, where genes related to phagocytosis and MHCII molecules were upregulated under heat stress conditions [40]. These findings suggest that *C. fuscus* exhibits the transcriptional activation of intracellular degradation pathways and antigen presentation-related genes under heat stress, which may play a role in neuronal homeostasis.

In summary, by regulating apoptosis-related pathways under stress conditions, *C. fuscus* may reduce the risk of large-scale neuronal loss. The coordinated action of lysosomes and phagosomes, along with microtubule-mediated phagosome maturation and its integration with antigen presentation, contributes to a comprehensive neuroprotective mechanism under heat stress. Following heat acclimation, *C. fuscus* exhibits an enhanced adaptive response, accelerating these processes to improve resilience. This adaptation not only facilitates the efficient removal of heat stress-induced cellular damage but strengthens immune responses, equipping the species with a robust survival strategy against extreme environmental fluctuations.

### 4.3. Cellular Energy-Saving Mode Suppresses Ribosomal Gene Expression and Reduces Protein Synthesis

The widespread anti-apoptotic response and active clearance of damaged cellular components in the *C. fuscus* brain entail high energy consumption [59]. To accommodate these metabolic demands, cells must strategically allocate resources, with ribosomal function likely playing a central role in this regulation. In our study, ribosome-related genes (RPL and RPS families) were highly expressed under normal temperature conditions (NT-C and HT-C), but were significantly downregulated following acute heat stress (NT-T72 and HT-T72), with a further decline during the recovery phase (NT-R72 and HT-R72). This pattern suggests that *C. fuscus* might adjust its protein synthesis strategy to cope with thermal stress. Similar ribosomal suppression has been observed in other fish species under heat stress. A similar suppression of ribosomal biogenesis has been observed in other fish species under heat stress. For example, in hybrid catfish (♀*Ictalurus punctatus* × ♂*Ictalurus furcatus*), high-temperature stress significantly reduced ribosomal biogenesis and overall protein synthesis, while simultaneously upregulating genes involved in protein folding and degradation to maintain cellular homeostasis [60]. Heat stress often triggers a cellular “energy-saving mode”, suppressing ribosomal gene expression to reduce protein synthesis and conserve energy, thereby enhancing cell survival. A comparable mechanism has been reported in rainbow trout (*Oncorhynchus mykiss*), where high-temperature stress downregulated ribosomal RNA expression while significantly upregulating heat shock proteins, suggesting a strategy to optimize energy utilization by reducing protein synthesis [61].

In the KEGG enrichment analysis of the T72 vs. C group in the NT condition, the endoplasmic reticulum (ER) protein processing pathway was significantly enriched. This finding is consistent with studies in silver pomfret (*Trachinotus ovatus*), where high-temperature exposure enhanced ER stress and suppressed ribosomal gene expression in the liver, reducing the accumulation of misfolded proteins [62]. A similar effect has been observed in rainbow trout (*Oncorhynchus mykiss*), where acute heat stress upregulated ER stress-related genes while simultaneously downregulating ribosomal genes, indicating a shift in cellular priorities from protein synthesis to stress mitigation [63]. Additionally, transcriptomic analyses of largemouth bass (*Micropterus salmoides*) revealed that heat stress suppressed protein translation machinery while enhancing microRNA-mediated post-transcriptional regulation, further supporting the energy conservation strategy through ribosomal suppression [10,15]. Interestingly, the degree of ribosomal gene downregulation was significantly lower in the HT group compared to the NT group. This suggests that heat-acclimated *C. fuscus* could better maintain basal protein synthesis during acute heat stress, ensuring fundamental biosynthetic support for neurons. This adaptive capacity may have been progressively established through long-term acclimation, allowing the HT group to more effectively balance cellular survival and protein synthesis under extreme environmental conditions. In Atlantic salmon (*Salmo salar*), long-term heat adaptation also improved ER stress responses and attenuated ribosomal suppression, boosting cellular resilience to acute heat stress [64]. In summary, our findings indicate that *C. fuscus* mitigates metabolic stress and energy expenditure by suppressing ribosome biogenesis and reducing de novo protein synthesis under heat stress conditions. However, the HT group exhibited a less pronounced decline in ribosomal gene expression than the NT group, suggesting that heat acclimation can alleviate ribosomal suppression. By maintaining a more stable level of basal protein synthesis under acute heat stress, heat-acclimated *C. fuscus* may have developed an adaptive advantage that facilitates a more effective balance between cellular survival and protein synthesis regulation in extreme thermal environments.

## 5. Conclusions

This study employed transcriptomic analysis to investigate the adaptive mechanisms in the brain of *C. fuscus* under different temperature treatments, focusing on the effects of heat acclimation and acute heat stress on neural responses. The results emphasized the critical role of CAMs and ECM in the initial heat stress response, stabilizing brain cell structures and activating downstream signaling pathways via integrins to promote cell survival and repair. Additionally, *C. fuscus* maintained neuronal viability and functioning through multiple mechanisms, including the inhibition of apoptosis, enhanced lysosome–phagosome interactions, and suppression of ribosome biogenesis. Heat-acclimated *C. fuscus* exhibited faster gene responses and enhanced tissue repair capacity, with increased resistance to apoptosis and a stronger immune response. These findings suggest that heat acclimation significantly improves the tolerance of *C. fuscus* to acute temperature fluctuations, offering valuable insights and theoretical support for understanding the adaptive strategies of aquaculture species in the face of global climate change.

## Data Availability

RNA-Seq data have been deposited in the NCBI database under the project accession number PRJNA1224955.

## Supplementary Materials

The following supporting information can be downloaded at: https://www.mdpi.com/article/10.3390/ani15091220/s1, Figure S1: Determination of the optimal number of clusters using the elbow method; Figure S2: Heatmap of differentially expressed genes (DEGs) across all treatment groups. Figure S3. GO and KEGG enrichment analysis of selected gene clusters after trend clustering in the NT group (A, B) and HT group (C, D). Table S1: Primers used for qRT-PCR in C. fuscus. Table S2: Quality control results of brain transcriptome sequencing data for the three treatment stages in the NT group of C. fuscus. Table S3: Quality control results of brain transcriptome sequencing data for the three treatment stages in the HT group of C. fuscus. Table S4: Differentially expressed genes (DEGs) between NT-T72 and NT-C groups. Table S5: Differentially expressed genes (DEGs) between NT-R72 and NT-C groups. Table S6: Differentially expressed genes (DEGs) between NT-R72 and NT-T72 groups. Table S7: Differentially expressed genes (DEGs) between HT-T72 and HT-C groups. Table S8: Differentially expressed genes (DEGs) between HT-R72 and HT-C groups. Table S9: Differentially expressed genes (DEGs) between HT-R72 and HT-T72 groups. Table S10: Supplementary information of genes highlighted in Figure 8.

## References

[1] Kiarsi M., Amiresmaili M., Mahmoodi M.R., Farahmandnia H., Nakhaee N., Zareiyan A., Aghababaeian H. (2023). Heat Waves and Adaptation: A Global Systematic Review. J. Therm. Biol..

[2] Xu W., Wang L. (2022). Editorial: The Physiological and Molecular Response of Aquatic Animals to Environmental Stresses. Front. Physiol..

[3] Ge G., Long Y., Song G., Li Q., Cui Z., Yan H. (2022). Transcriptomic Profiling Revealed Signaling Pathways Associated with the Spawning of Female Zebrafish under Cold Stress. Int. J. Mol. Sci..

[4] Luo L., Zhao Z., Zhang R., Guo K., Wang S., Xu W., Wang C. (2022). The Effects of Temperature Changes on the Isozyme and Hsp70 Levels of the Amur Sturgeon, *Acipenser Schrenckii*, at Two Acclimation Temperatures. Aquaculture.

[5] Li S., Liu Y., Li B., Ding L., Wei X., Wang P., Chen Z., Han S., Huang T., Wang B. (2022). Physiological Responses to Heat Stress in the Liver of Rainbow Trout (*Oncorhynchus mykiss*) Revealed by UPLC-QTOF-MS Metabolomics and Biochemical Assays. Ecotoxicol. Environ. Saf..

[6] Maulu S., Hasimuna O.J., Haambiya L.H., Monde C., Musuka C.G., Makorwa T.H., Munganga B.P., Phiri K.J., Nsekanabo J.D. (2021). Climate Change Effects on Aquaculture Production: Sustainability Implications, Mitigation, and Adaptations. Front. Sustain. Food Syst..

[7] Cheng X., Li F., Lu J., Wen Y., Li Z., Liao J., Cao J., He X., Sun J., Liu Q. (2024). Transcriptome Analysis in Gill Reveals the Adaptive Mechanism of Domesticated Common Carp to the High Temperature in Shallow Rice Paddies. Aquaculture.

[8] Nonnis S., Angiulli E., Maffioli E., Frabetti F., Negri A., Cioni C., Alleva E., Romeo V., Tedeschi G., Toni M. (2021). Acute Environmental Temperature Variation Affects Brain Protein Expression, Anxiety and Explorative Behaviour in Adult Zebrafish. Sci. Rep..

[9] Haesemeyer M., Robson D.N., Li J.M., Schier A.F., Engert F. (2018). A Brain-Wide Circuit Model of Heat-Evoked Swimming Behavior in Larval Zebrafish. Neuron.

[10] Zhao Y., Duan M., Lin X., Li W., Liu H., Meng K., Liu F., Hu W., Luo D. (2024). Molecular Underpinnings Underlying Behaviors Changes in the Brain of Juvenile Common Carp (*Cyrinus carpio*) in Response to Warming. J. Adv. Res..

[11] Zhang W., Xu X., Li J., Shen Y. (2022). Transcriptomic Analysis of the Liver and Brain in Grass Carp (*Ctenopharyngodon idella*) Under Heat Stress. Mar. Biotechnol..

[12] Benítez-Dorta V., Caballero M.J., Betancor M.B., Manchado M., Tort L., Torrecillas S., Zamorano M.J., Izquierdo M., Montero D. (2017). Effects of Thermal Stress on the Expression of Glucocorticoid Receptor Complex Linked Genes in Senegalese Sole (*Solea senegalensis*): Acute and Adaptive Stress Responses. Gen. Comp. Endocrinol..

[13] Yang Q., Zhong Y., Yang F., Li H., Tran N.T., Zhang M., Wang L., He L., Zhang Z., Ge H. (2024). Transcriptome Analysis of Sea Cucumber (*Apostichopus japonicus*) in Southern China under Heat Stress. Aquac. Rep..

[14] Yang S., Zhao T., Ma A., Huang Z., Liu Z., Cui W., Zhang J., Zhu C., Guo X., Yuan C. (2020). Metabolic Responses in *Scophthalmus Maximus* Kidney Subjected to Thermal Stress. Fish Shellfish Immunol..

[15] Zhao T., Ma A., Huang Z., Liu Z., Sun Z., Zhu C., Yang J., Li Y., Wang Q., Qiao X. (2021). Transcriptome Analysis Reveals That High Temperatures Alter Modes of Lipid Metabolism in Juvenile Turbot (*Scophthalmus maximus*) Liver. Comp. Biochem. Physiol. Part D Genom. Proteom..

[16] Li Q., Xiong L., Zhu Y., Zheng A., Zheng S. (2024). Effects of Acute Temperature Stress on the Expression of Related Genes in the Brain of *Opsariichthys bidens*. Fishes.

[17] Lyu L., Wen H., Li Y., Li J., Zhao J., Zhang S., Song M., Wang X. (2018). Deep Transcriptomic Analysis of Black Rockfish (*Sebastes schlegelii*) Provides New Insights on Responses to Acute Temperature Stress. Sci. Rep..

[18] Tian C.-X., Lin X.-H., Zhou D.-Y., Chen Y., Shen Y.-J., Ye M.-H., Duan C.-Y., Zhang Y.-L., Yang B.-L., Deng S.-P. (2023). A Chromosome-Level Genome Assembly of Hong Kong Catfish (*Clarias fuscus*) Uncovers a Sex-Determining Region. BMC Genom..

[19] Anderson M.J., Fast A.W. (1991). Temperature and Feed Rate Effects on Chinese Catfish, *Clarias fuscus* (Lacepède), Growth. Aquac. Res..

[20] Liu Y., Tian C., Yang Z., Huang C., Jiao K., Yang L., Duan C., Zhang Z., Li G. (2024). Effects of Chronic Heat Stress on Growth, Apoptosis, Antioxidant Enzymes, Transcriptomic Profiles, and Immune-Related Genes of Hong Kong Catfish (*Clarias fuscus*). Animals.

[21] Duan C., Tian C., Guan Y., Xu H., Yang L., Chen Y., Liu Y., Shen Y., Zhang Y., Cao S. (2024). Long-Term Thermal Stress Induces Hepatic Injury and Alters the Thermotolerance Response in Hong Kong Catfish (*Clarias fuscus*). Aquaculture.

[22] Duan C., Zhou D., Feng R., Li X., Yang L., Li X., Li G., Chen H., Liao Y., Tian C. (2024). Long-Term Thermal Acclimation Enhances Heat Resistance of Hong Kong Catfish (*Clarias fuscus*) by Modulating Gill Tissue Structure, Antioxidant Capacity and Immune Metabolic Pathways. Ecotoxicol. Environ. Saf..

[23] Duan C., Yang L., Chen W., Zhou D., Cao S., Zhang Y., Li G., Chen H., Tian C. (2025). Long-Term Thermal Stress Reshapes the Tolerance of Head Kidney of Hong Kong Catfish (*Clarias fuscus*) to Acute Heat Shock by Regulating Energy Metabolism and Immune Response. Comp. Biochem. Physiol. Part D Genom. Proteom..

[24] Lin X., Tan J., Shen Y., Yang B., Zhang Y., Liao Y., Wang P., Zhou D., Li G., Tian C. (2022). A High-Density Genetic Linkage Map and QTL Mapping for Sex in *Clarias Fuscus*. Aquaculture.

[25] Garber M., Grabherr M.G., Guttman M., Trapnell C. (2011). Computational Methods for Transcriptome Annotation and Quantification Using RNA-Seq. Nat. Methods.

[26] Shen Y., Zhu Y., Yang Z., Ye M., Zhou D., Li G., Tian C. (2024). Screening for qRT-PCR Internal Reference Genes in *Clarias Fuscus*. Prog. Fish. Sci..

[27] Takeichi M. (2007). The Cadherin Superfamily in Neuronal Connections and Interactions. Nat. Rev. Neurosci..

[28] Huang Z., Ma A., Yang S., Liu X., Zhao T., Zhang J., Wang X., Sun Z., Liu Z., Xu R. (2020). Transcriptome Analysis and Weighted Gene Co-Expression Network Reveals Potential Genes Responses to Heat Stress in Turbot *Scophthalmus Maximus*. Comp. Biochem. Physiol. Part D Genom. Proteom..

[29] Kraitavin W., Yoshitake K., Igarashi Y., Mitsuyama S., Kinoshita S., Kambayashi D., Watabe S., Asakawa S. (2019). Transcriptome Analysis of Yamame (*Oncorhynchus masou*) in Normal Conditions after Heat Stress. Biology.

[30] Brusés J.L. (2006). N-Cadherin Signaling in Synapse Formation and Neuronal Physiology. Mol. Neurobiol..

[31] Basu R., Taylor M.R., Williams M.E. (2015). The Classic Cadherins in Synaptic Specificity. Cell Adhes. Migr..

[32] Dityatev A., Bukalo O., Schachner M. (2008). Modulation of Synaptic Transmission and Plasticity by Cell Adhesion and Repulsion Molecules. Neuron Glia Biol..

[33] Faivre-Sarrailh C., Devaux J.J. (2013). Neuro-Glial Interactions at the Nodes of Ranvier: Implication in Health and Diseases. Front. Cell. Neurosci..

[34] Belluardo N., White T.W., Srinivas M., Trovato-Salinaro A., Ripps H., Mudò G., Bruzzone R., Condorelli D.F. (2001). Identification and Functional Expression of HCx31.9, a Novel Gap Junction Gene. Cell Commun. Adhes..

[35] López-Bendito G., Molnár Z. (2003). Thalamocortical Development: How Are We Going to Get There?. Nat. Rev. Neurosci..

[36] Takai Y., Miyoshi J., Ikeda W., Ogita H. (2008). Nectins and Nectin-like Molecules: Roles in Contact Inhibition of Cell Movement and Proliferation. Nat. Rev. Mol. Cell Biol..

[37] Südhof T.C. (2017). Synaptic Neurexin Complexes: A Molecular Code for the Logic of Neural Circuits. Cell.

[38] Maness P.F., Schachner M. (2007). Neural Recognition Molecules of the Immunoglobulin Superfamily: Signaling Transducers of Axon Guidance and Neuronal Migration. Nat. Neurosci..

[39] Hynes R.O. (2002). Integrins: Bidirectional, Allosteric Signaling Machines. Cell.

[40] Yang Y., Yu H., Li H., Wang A., Yu H. (2016). Effect of High Temperature on Immune Response of Grass Carp (*Ctenopharyngodon idellus*) by Transcriptome Analysis. Fish Shellfish Immunol..

[41] Cheng C.-H., Yang F.-F., Ling R.-Z., Liao S.-A., Miao Y.-T., Ye C.-X., Wang A.-L. (2015). Effects of Ammonia Exposure on Apoptosis, Oxidative Stress and Immune Response in Pufferfish (*Takifugu obscurus*). Aquat. Toxicol..

[42] Liu E., Zhao X., Li C., Wang Y., Li L., Zhu H., Ling Q. (2022). Effects of Acute Heat Stress on Liver Damage, Apoptosis and Inflammation of Pikeperch (*Sander lucioperca*). J. Therm. Biol..

[43] Fleisch V.C., Fraser B., Allison W.T. (2011). Investigating Regeneration and Functional Integration of CNS Neurons: Lessons from Zebrafish Genetics and Other Fish Species. Biochim. Biophys. Acta BBA Mol. Basis Dis..

[44] Tan C., Pang X., Zhang J., Yan C., Xu Z., Shao W., Wu J., Li Y., Du X., Yang S. (2024). Effects of Chronic Heat Stress on Spleen Structure, Apoptosis and Immune Response in Siberian Sturgeon (*Acipenser baerii*). Isr. J. Aquac. Bamidgeh.

[45] Cavalcante G.C., Schaan A.P., Cabral G.F., Santana-da-Silva M.N., Pinto P., Vidal A.F., Ribeiro-dos-Santos Â. (2019). A Cell’s Fate: An Overview of the Molecular Biology and Genetics of Apoptosis. Int. J. Mol. Sci..

[46] Yu S., Chen L., Song K., Shu T., Fang Z., Ding L., Liu J., Jiang L., Zhang G., Zhang B. (2022). Irreversible Electroporation Mediates Glioma Apoptosis via Upregulation of AP-1 and Bim: Transcriptome Evidence. Brain Sci..

[47] Yabu T., Shiba H., Shibasaki Y., Nakanishi T., Imamura S., Touhata K., Yamashita M. (2015). Stress-Induced Ceramide Generation and Apoptosis via the Phosphorylation and Activation of nSMase1 by JNK Signaling. Cell Death Differ..

[48] Wang Y., Su C., Liu Q., Hao X., Han S., Doretto L.B., Rosa I.F., Yang Y., Shao C., Wang Q. (2023). Transcriptome Analysis Revealed the Early Heat Stress Response in the Brain of Chinese Tongue Sole (*Cynoglossus semilaevis*). Animals.

[49] Liu M., Zhou Y., Guo X., Wei W., Li Z., Zhou L., Wang Z., Gui J. (2022). Comparative Transcriptomes and Metabolomes Reveal Different Tolerance Mechanisms to Cold Stress in Two Different Catfish Species. Aquaculture.

[50] Jing Z., Chen Q., Yan C., Zhang C., Xu Z., Huang X., Wu J., Li Y., Yang S. (2023). Effects of Chronic Heat Stress on Kidney Damage, Apoptosis, Inflammation, and Heat Shock Proteins of Siberian Sturgeon (*Acipenser baerii*). Animals.

[51] Han P., Yuan M., Sun Z., Xue Y., Liu X., Chen J., Yu H., Wang X. (2025). Effect of Heat Exposure on Histology, Transcriptomics and Co-Expression Network: A Synthetic Study in Japanese Flounder (*Paralichthys olivaceus*). Aquaculture.

[52] Logan C.A., Somero G.N. (2011). Effects of Thermal Acclimation on Transcriptional Responses to Acute Heat Stress in the Eurythermal Fish *Gillichthys mirabilis* (Cooper). Am. J. Physiol. Regul. Integr. Comp. Physiol..

[53] Lin H., Ao H., Guo G., Liu M. (2023). The Role and Mechanism of Metformin in Inflammatory Diseases. J. Inflamm. Res..

[54] Wang Y., Li C., Pan C., Liu E., Zhao X., Ling Q. (2019). Alterations to Transcriptomic Profile, Histopathology, and Oxidative Stress in Liver of Pikeperch (*Sander lucioperca*) under Heat Stress. Fish Shellfish Immunol..

[55] Beemelmanns A., Zanuzzo F.S., Xue X., Sandrelli R.M., Rise M.L., Gamperl A.K. (2021). The Transcriptomic Responses of Atlantic Salmon (*Salmo Salar*) to High Temperature Stress Alone, and in Combination with Moderate Hypoxia. BMC Genom..

[56] Mostowy S., Shenoy A.R. (2015). The Cytoskeleton in Cell-Autonomous Immunity: Structural Determinants of Host Defence. Nat. Rev. Immunol..

[57] Nair-Gupta P., Baccarini A., Tung N., Seyffer F., Florey O., Huang Y., Banerjee M., Overholtzer M., Roche P.A., Tampé R. (2014). TLR Signals Induce Phagosomal MHC-I Delivery from the Endosomal Recycling Compartment to Allow Cross-Presentation. Cell.

[58] Münz C. (2021). The Macroautophagy Machinery in MHC Restricted Antigen Presentation. Front. Immunol..

[59] Jiao C., Zou J., Chen Z., Zheng F., Xu Z., Lin Y.-H., Wang Q. (2021). Dietary Glutamine Inclusion Regulates Immune and Antioxidant System, as Well as Programmed Cell Death in Fish to Protect against *Flavobacterium Columnare* Infection. Antioxidants.

[60] Liu S., Wang X., Sun F., Zhang J., Feng J., Liu H., Rajendran K.V., Sun L., Zhang Y., Jiang Y. (2013). RNA-Seq Reveals Expression Signatures of Genes Involved in Oxygen Transport, Protein Synthesis, Folding, and Degradation in Response to Heat Stress in Catfish. Physiol. Genom..

[61] Quan J., Kang Y., Luo Z., Zhao G., Ma F., Li L., Liu Z. (2020). Identification and Characterization of Long Noncoding RNAs Provide Insight into the Regulation of Gene Expression in Response to Heat Stress in Rainbow Trout (*Oncorhynchus mykiss*). Comp. Biochem. Physiol. Part D Genom. Proteom..

[62] Li Q.-Q., Zhang J., Wang H.-Y., Niu S.-F., Wu R.-X., Tang B.-G., Wang Q.-H., Liang Z.-B., Liang Y.-S. (2023). Transcriptomic Response of the Liver Tissue in *Trachinotus ovatus* to Acute Heat Stress. Animals.

[63] Zhou C.-Q., Ka W., Zhang H.-J., Li Y.-L., Gao P., Long R.-J., Yang S.-W., Wang J.-L. (2022). RNA-Seq Analysis of the Key Long Noncoding RNAs and mRNAs Related to the Regulation of Acute Heat Stress in Rainbow Trout. Animals.

[64] Ge K., Fan Z., Huang T., Gu W., Wang G., Liu E., Pan R., Li D., Sun Y., Yao Z. (2024). Influence of Increasing Acclimation Temperature on Growth, Digestion, Antioxidant Capacity, Liver Transcriptome and Intestinal Microflora of Ussruri Whitefish *Coregonus Ussuriensis* Berg. Fish Shellfish Immunol..

