# Supplementary material for: Heat Acclimation Enhances Brain Resilience to Acute Thermal Stress in Clarias fuscus by Modulating Cell Adhesion, Anti-Apoptotic Pathways, and Intracellular Degradation Mechanisms
Source: Animals (Basel). 2025 Apr 25;15(9):1220. doi: 10.3390/ani15091220 (PMC12071039; doi:10.3390/ani15091220)
Supplement: Supplementary file 1 [file animals-15-01220-s001.zip › Figure S1.pdf]

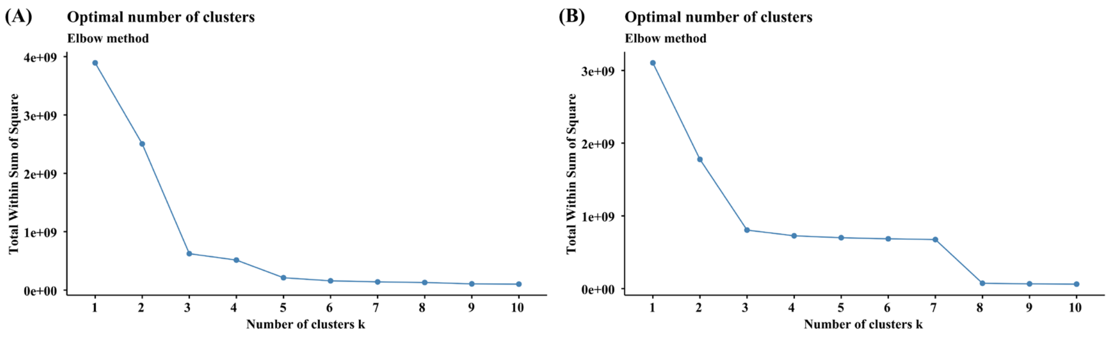

Figure S1. Determination of the optimal number of clusters using the elbow method. The x-axis represents the number of clusters (k), while the y-axis indicates the total within-cluster sum of squares (WSS) for each cluster number.(A) Elbow plot for DEGs in the NT group.(B) Elbow plot for DEGs in the HT group.
