# Supplementary material for: Heat Acclimation Enhances Brain Resilience to Acute Thermal Stress in Clarias fuscus by Modulating Cell Adhesion, Anti-Apoptotic Pathways, and Intracellular Degradation Mechanisms
Source: Animals (Basel). 2025 Apr 25;15(9):1220. doi: 10.3390/ani15091220 (PMC12071039; doi:10.3390/ani15091220)
Supplement: Supplementary file 1 [file animals-15-01220-s001.zip › Figure S3.pdf]

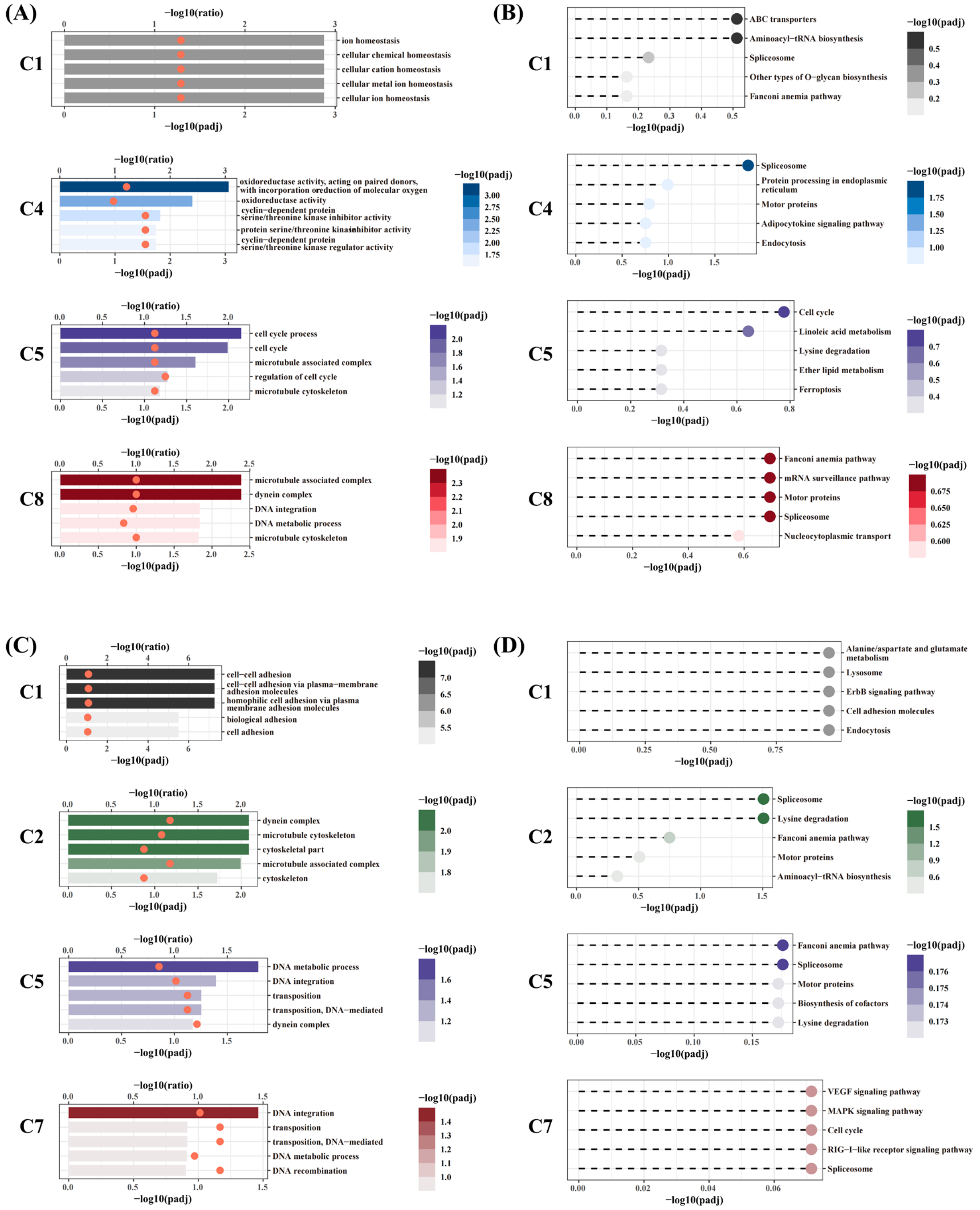

Figure S3. GO and KEGG enrichment analysis of selected gene clusters after trend clustering in the NT group (A, B) and HT group (C, D). (A, C) GO functional enrichment analysis. The y-axis represents GO terms. The upper x-axis indicates -log10(ratio), with its value represented by the position of the orange dots. The lower x-axis indicates -log10(padj), with bar color intensity reflecting enrichment significance, where darker colors denote higher significance. (B, D) KEGG pathway enrichment analysis. The color intensity of the dots represents -log10(padj), with darker colors indicating higher enrichment significance.
