# Supplementary material for: Heat Acclimation Enhances Brain Resilience to Acute Thermal Stress in Clarias fuscus by Modulating Cell Adhesion, Anti-Apoptotic Pathways, and Intracellular Degradation Mechanisms
Source: Animals (Basel). 2025 Apr 25;15(9):1220. doi: 10.3390/ani15091220 (PMC12071039; doi:10.3390/ani15091220)
Supplement: Supplementary file 1 [file animals-15-01220-s001.zip › Table S1.pdf]

Table S1. Primers used for qRT-PCR in *C. fuscus*.

Gene name	Accession number	Primer sequences(5′-3′)
canx	Cfu0039080	F:TGAGGAAATCGCCAAGT<br>R:TGAAGGGTCGCAGTAGG
tgfbr2	Cfu0074480	F:AAGACCACGACAACAAGAAG<br>R:GAGGATGACGGTAAGAACG
cript	Cfu0002840	F:AGAGTGGAGGACGGAAGC<br>R:CCCAGATTGGTGGACAGA
apoh	Cfu0021310	F:TTTGGGAATCGCAGAGG<br>R:GGTGACAGCCGTATTTGACT
cryab	Cfu0165010	F: TTGCCAACCTATCCTTCG<br>R: TCCGCTGACCTTTACTCG
mbp	Cfu0125190	F: GCGATAAGAAGCGGAAGAG<br>R: GGGCAGGAGACACGATAGA
atp5g3	Cfu0022560	F:TCGGATACGCCAGGAAC<br>R:AAGCGACCATCAAACAGAA
fabp4	Cfu0106210	F:TGACAGAAAGACCACGACC<br>R:CCTCACTGCCACCACATC
tubb4b	Cfu0062730	F: GCTATTCCGCCGTATCTC<br>R: CAGCCTCGGTGAACTCC
actb2	Cfu0180250	F: TGAAGGACATCCGCCGTGGTAA<br>R: GGCAATGTGAGCAGTGTGACAGT
