# Supplementary material for: Heat Acclimation Enhances Brain Resilience to Acute Thermal Stress in Clarias fuscus by Modulating Cell Adhesion, Anti-Apoptotic Pathways, and Intracellular Degradation Mechanisms
Source: Animals (Basel). 2025 Apr 25;15(9):1220. doi: 10.3390/ani15091220 (PMC12071039; doi:10.3390/ani15091220)
Supplement: Supplementary file 1 [file animals-15-01220-s001.zip › Table S2.pdf]

Table S2. Quality control results of brain transcriptome sequencing data for the three treatment stages in the NT group of *C. fuscus*.

	C-1	C-2	C-3	T72-1	T72-2	T72-3	R72-1	R72-2	R72-3
**Raw reads**	48877970	46139384	45099818	47022306	46603088	49361708	55974106	46956116	41187130
**Clean reads**	47417476	44951646	42310080	45982418	45692716	47848808	54391062	45556406	39096830
**Clean bases (G)**	7.11G	6.74G	6.35G	6.9G	6.85G	7.18G	8.16G	6.83G	5.86G
**Q20（%）**	96.55	96.93	96.39	96.56	96.68	96.84	97.3	97.22	96.8
**Q30（%）**	91.71	92.33	91.67	91.7	91.86	92.17	92.94	92.86	91.96
**GC pct（%）**	41.31	43.56	45.97	43.49	43.24	43.18	45.2	45.9	46.39
**Total Mapped**	39583492 (83.48%)	38432912 (85.5%)	30689089 (72.53%)	29656259 (64.49%)	37052602 (81.09%)	39802244 (83.18%)	47686785 (87.67%)	40303530 (88.47%)	34645506 (88.61%)
**Uniq Mapped Reads**	39095739 (82.45%)	37924855 (84.37%)	29979095 (70.86%)	29066135 (63.21%)	36481594 (79.84%)	39245407 (82.02%)	46832887 (86.1%)	39434611 (86.56%)	34076241 (87.16%)
**Multiple Mapped Reads**	487753 (1.03%)	508057 (1.13%)	709994 (1.68%)	590124 (1.28%)	571008 (1.25%)	556837 (1.16%)	853898 (1.57%)	868919 (1.91%)	569265 (1.46%)
