# Supplementary material for: Heat Acclimation Enhances Brain Resilience to Acute Thermal Stress in Clarias fuscus by Modulating Cell Adhesion, Anti-Apoptotic Pathways, and Intracellular Degradation Mechanisms
Source: Animals (Basel). 2025 Apr 25;15(9):1220. doi: 10.3390/ani15091220 (PMC12071039; doi:10.3390/ani15091220)
Supplement: Supplementary file 1 [file animals-15-01220-s001.zip › Table S3.pdf]

Table S3. Quality control results of brain transcriptome sequencing data for the three treatment stages in the HT group of *C. fuscus*.

	C-1	C-2	C-3	T72-1	T72-2	T72-3	R72-1	R72-2	R72-3
**Raw reads**	44091880	46958978	39275842	46783252	46310654	46155506	55501424	48989730	46407478
**Clean reads**	42667890	45789006	38316990	45980412	45141474	45074152	53383510	46750246	44329342
**Clean bases (G)**	6.4G	6.87G	5.75G	6.9G	6.77G	6.76G	8.01G	7.01G	6.65G
**Q20（%）**	96.52	96.45	96.57	96.67	96.74	96.63	97.56	97.44	97.31
**Q30（%）**	91.5	91.43	91.71	91.77	91.89	91.72	93.52	93.27	93.02
**GC pct（%）**	42.49	42.29	42.77	42.86	43.82	43.22	47.19	47.48	47.26
**Total Mapped**	35290844 (82.71%)	36508012 (79.73%)	29797708 (77.77%)	39566755 (86.05%)	36836515 (81.6%)	36290634 (80.51%)	47602975 (89.17%)	42096822 (90.05%)	39302680 (88.66%)
**Uniq Mapped Reads**	34822826 (81.61%)	35931720 (78.47%)	29209802 (76.23%)	39038653 (84.9%)	36265195 (80.34%)	35687664 (79.18%)	46608302 (87.31%)	41203890 (88.14%)	38609345 (87.1%)
**Multiple Mapped Reads**	468018 (1.1%)	576292 (1.26%)	587906 (1.53%)	528102 (1.15%)	571320 (1.27%)	602970 (1.34%)	994673 (1.86%)	892932 (1.91%)	693335 (1.56%)
