# Supplementary figures and images for: Heat Acclimation Enhances Brain Resilience to Acute Thermal Stress in Clarias fuscus by Modulating Cell Adhesion, Anti-Apoptotic Pathways, and Intracellular Degradation Mechanisms
Source: Animals (Basel). 2025 Apr 25;15(9):1220. doi: 10.3390/ani15091220 (PMC12071039; doi:10.3390/ani15091220)

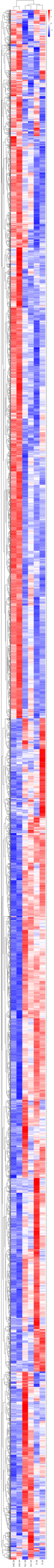

Supplement: Supplementary file 1 [file animals-15-01220-s001.zip › Figure S2.pdf]
